# Supplementary material for: Reconciling Mining with the Conservation of Cave Biodiversity: A Quantitative Baseline to Help Establish Conservation Priorities
Source: PLoS One. 2016 Dec 20;11(12):e0168348. doi: 10.1371/journal.pone.0168348 (PMC5173368; doi:10.1371/journal.pone.0168348)
Supplement: S1 Dataset — (ZIP) [file pone.0168348.s002.zip › Taxa/Serra Norte/SN_2013/N4E/Lista N4E-83.pdf]

CAVIDADE N4E-0083

Classe	Ordem	Fam/Outros	Gên/Outros	Espécie	S-U	U-S	Zon.
Annelida	Oligochaeta			jovem	1	5	E
Arachnida	Acari	(Parasitengonina)		sp.5		X	E
Arachnida	Acari	Laelapidae		sp.71	X		E
Arachnida	Acari	Neothyridae	*Diplothyrus*	jovem		X	E
Arachnida	Acari	Prostigmata		sp.3		X	E
Arachnida	Amblypygi	Charinidae	*Charinus*	sp.1	1		E
Arachnida	Amblypygi	Phrynidae	*Heterophrynus*	*longicornis*	3	1	E
Arachnida	Araneae	Araneidae	*Alpaida*	*antonio*	X	X	E
Arachnida	Araneae	Barychelidae		jovem	1		E
Arachnida	Araneae	Ctenidae	*Ctenus*	sp.1	2		E
Arachnida	Araneae	Ochyroceratidae		jovem	X		E
Arachnida	Araneae	Oonopidae	aff. *Xyccarph*	sp.2		X	E
Arachnida	Araneae	Oonopidae	aff. *Xyccarph*	sp.3	X		E
Arachnida	Araneae	Pholcidae	*Leptopholcus*	sp.1	X		E
Arachnida	Araneae	Pholcidae	*Mesabolivar*	*aurantiacus*	X	X	E
Arachnida	Araneae	Pholcidae	*Mesabolivar*	sp.1	X	X	E
Arachnida	Araneae	Prodidomidae	*Lygromma*	sp.2		X	E
Arachnida	Araneae	Salticidae		sp.9	X		E
Arachnida	Araneae	Scytodidae	*Scytodes*	*eleonorae*		1	E
Arachnida	Araneae	Theridiosomatidae	*Plato*	sp.1		X	E
Arachnida	Opiliones	Cosmetidae	*Roquettea*	*singularis*	3		E
Arachnida	Opiliones	Stygnidae	*Protimesius*	*gracilis*	3	1	E
Arachnida	Schizomida	Hubbardiidae	aff. *Rowlandius*	sp.1		X	E
Chilopoda	Geophilomorpha	Geophilidae		sp.1		1	E
Chilopoda	Scolopendromorpha	Cryptopidae	*Cryptops*	jovem		2	E
Diplopoda	Glomeridesmida	Glomeridesmidae		sp.1		1	E
Diplopoda	Polydesmida	Cyrtodesmidae		sp.1	X		E
Diplopoda	Polydesmida	Fuhrmannodesmidae		sp.1	X	X	E
Diplopoda	Polydesmida	Paradoxosomatidae		sp.1		1	E
Diplopoda	Spirostreptida	Pseudonannolenidae	*Pseudonannolene*	jovem	1		
Entognatha	Collembola	Paronellidae		sp.1		X	E
Entognatha	Collembola	Poduromorpha		jovem		X	E
Entognatha	Diplura	Campodeidae		sp.1	X		E
Insecta	Coleoptera	Carabidae		sp.3		X	E
Insecta	Diptera	Cecidomyiidae	Cecidomyiinae	sp.		X	E
Insecta	Diptera	Psychodidae	Phlebotominae	sp.	X	X	E
Insecta	Diptera	Tipulidae	Tipulinae	sp.	X	X	E
Insecta	Heteroptera	Reduviidae	Reduviinae	sp.	1		E
Insecta	Homoptera	Cixiidae		jovem	X	X	E
Insecta	Hymenoptera	Formicidae	*Camponotus*	sp.2	X	X	E
Insecta	Hymenoptera	Formicidae	*Pachycondyla*	sp.2	X	X	E
Insecta	Hymenoptera	Formicidae	*Paratrechina*	sp.1	X	X	E
Insecta	Hymenoptera	Formicidae	*Pheidole*	sp.2	X		E
Insecta	Hymenoptera	Formicidae	*Solenopsis*	sp.1	X		E
Insecta	Isoptera	Termitidae	*Nasutitermes*	sp.	X	X	E
Insecta	Lepidoptera	Noctuoidea		sp.7		1	E
Insecta	Orthoptera	Gryllidae	Nemobiinae	jovem		1	E
Insecta	Orthoptera	Phalangopsidae	*Paraclodes*	sp.	3		E
Insecta	Orthoptera	Phalangopsidae	*Phalangopsis*	sp.	10	6	E

Gastropoda	Pulmonata	Subulinidae		sp.5		X	E
Gastropoda	Pulmonata	Systrophiidae	*Happia*	sp.	X	X	E
Malacostraca	Isopoda	Philosciidae		sp.1		X	E
Malacostraca	Isopoda	Philosciidae		sp.2		X	E
Malacostraca	Isopoda	Scleropactidae		sp.1		X	E
Amphibia	Anura	Strabomantidae	*Pristimantis*	cf. *fenestratus*	5		E
Mammalia	Chiroptera	Emballonuridae	*Peropteryx*	*kappleri*	3	10	E
Mammalia	Chiroptera	Furipteridae	*Furipterus*	*horrens*		20	E
Mammalia	Chiroptera	Phyllostomidae	*Carollia*	*perspicillata*		10	E
Reptilia	Squamata	Gekkonidae	*Thecadactylus*	sp.	1	1	E
